# Supplementary figures and images for: Cost-effective sequence analysis of 113 genes in 1,192 probands with retinitis pigmentosa and Leber congenital amaurosis
Source: Front Cell Dev Biol. 2023 Feb 3;11:1112270. doi: 10.3389/fcell.2023.1112270 (PMC9936074; doi:10.3389/fcell.2023.1112270)

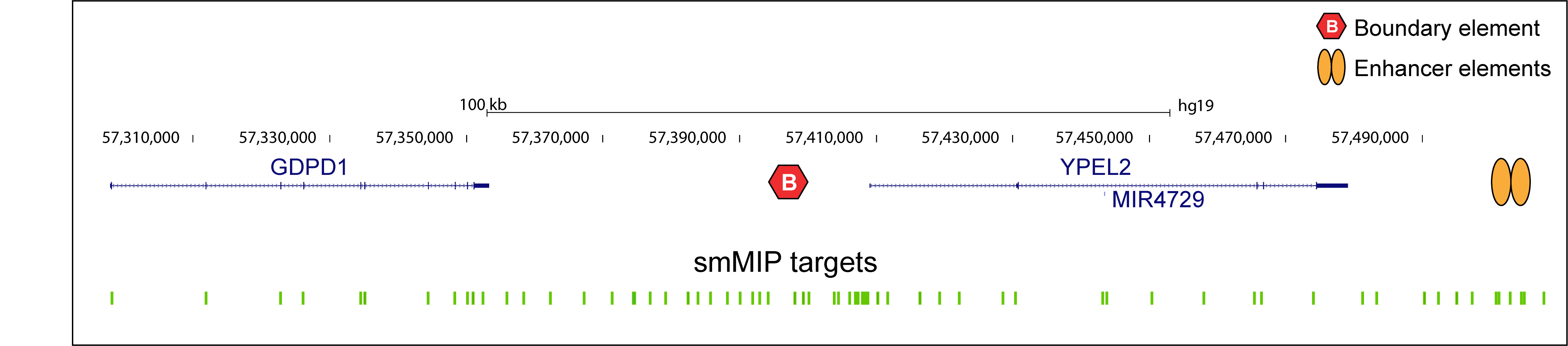

Supplement: Supplementary file 4 [file Image1.TIF]
